# Supplementary material for: Using specialist screening practitioners (SSPs) to increase uptake of bowel scope (flexible sigmoidoscopy) screening: results of a feasibility single-stage phase II randomised trial
Source: BMJ Open. 2019 Feb 15;9(2):e023801. doi: 10.1136/bmjopen-2018-023801 (PMC6398706; doi:10.1136/bmjopen-2018-023801)
Supplement: Supplementary data [file bmjopen-2018-023801supp001.pdf]

‘NO THANK YOU’

Please tell us why you do not wish to take part in this study? (*Please tick all that apply*)

- ☐ I do not fully understand what the study is about
- ☐ I do not have the time to read, complete and return the consent slip
- ☐ I do not want to share my personal details with the researchers
- ☐ I do not want to receive additional phone calls from the screening centre
- ☐ I do not want any phone calls from the screening centre to be recorded
- ☐ I do not think I will need any support.
- ☐ I know I am not eligible for bowel scope screening
- ☐ I have already decided **NOT** to have the bowel scope screening test

  If this is the reason, could you briefly tell us why you do not want to have the test:

  ………………………………………………………………………………………………..

  ………………………………………………………………………………………………..
- ☐ Other (please specify): I do not wish to take part because…………………………………………

  ………………………………………………………………………………………………..

  ………………………………………………………………………………………………..

  ………………………………………………………………………………………………..

FREEPOST

University College London

Gower Street

London
